# Prevalence of *Bacillus cereus* in dairy powders focusing on its toxigenic genes and antimicrobial resistance

**DOI:** 10.1007/s00203-022-02945-3

**Published:** 2022-05-19

**Authors:** Aml S. Ibrahim, Nagah M. Hafiz, M. F. Saad

**Affiliations:** Department of Food Hygiene and Control, Faculty of Veterinary Medicine, Cairo University, Giza, Egypt

**Keywords:** *Bacillus cereus*, Powdered milk, Powdered infant milk formula, Milk–Cereal-based infant formula, Toxigenic genes

## Abstract

*Bacillus cereus* is a common environmental foodborne microorganism that is mainly found to harbor toxigenic genes with multiple antibiotic resistances and is linked to threatening the safety of dried milk in concern to powdered infant milk formula. In the current investigation, the mean value of *B. cereus* in 140 samples of powdered milk was 0.57 × 10^2^ ± 0.182 × 10^2^, 0.15 × 10^2^ ± 0.027 × 10^2^, 0.21 × 10^2^ ± 0.035 × 10^2^, and 0.32 × 10^2^ ± 0.072 × 10^2^ CFU/g in a percentage of 64.0 samples of whole milk powder, 43.3 of skim milk powder, 26.7 of powdered infant milk formula and 36.7 milk–cereal-based infant formula, respectively. The results revealed that *B. cereus* isolates were found to harbor toxigenic genes in the following percentages: 77.8, 2.0, 72.7, 16.2, and 67.7 for *nhe*, *hbl*, *cytK*, *ces*, and *bceT*, respectively. Despite all evaluated *B. cereus* strains were originated from dairy powders, they showed a significant difference (*P* < 0.05) in their harbored toxigenic *cytK* gene between whole and skim milk powders with powdered infant formula and milk–cereal-based infant formula, as well as between powdered infant formula and milk–cereal-based infant formula. All isolated *B. cereus* strains were resistant to cefoxitin, colistin sulfate, neomycin, trimethoprim–sulfamethoxazole, oxacillin, and penicillin. Based on the antimicrobial resistance of *B. cereus* strains to cephalothin, chloramphenicol, nalidixic acid, and tetracycline, there was a significant difference (*P* < 0.05) between powdered infant milk formula and whole milk powder strains. This survey is one of few studies proceeded in Egypt to determine the prevalence of toxigenic *B. cereus* strains in milk–cereal-based infant formula and powdered infant formula as well as skim milk powder.

**Supplementary Information:**

The online version contains supplementary material available at 10.1007/s00203-022-02945-3.

## Introduction

Dairy powders are a prevalent product due to their extended usable time with multilateral kind. Infants are massive consumers of powdered milk-based products, as they may potentially in any way be with a weak immune system, especially newborns that did not receive breastfeeding; the passive immunity was not being transferred to them. Baby foods are well known to be the significant nutrition source of choice for kids, especially in the first part of life when they cannot digest other complex food. Their high values for proteins, minerals, fats, and vitamins are undeniable. Infants and babies have the weakest immune system, so the safety and hygienic quality of these baby foods are highly significant in avoiding and controlling their microbial contamination (Rahimi et al. 2013; Sadek et al. 2018).

As declared by the World Health Organization (WHO 2007), *B. cereus* has now become one of the widespread bacteria that subsist even in pasteurized food products and is categorized as group C that distinguished less danger with the possibility to cause outbreaks in infants by eating infant formula. *B. cereus *is the most recurrent bacterial contaminant in powdered milk products especially dried infant formula (Di Pinto et al. 2013; Sadek et al. 2018).

The spores of *B. cereus* are capable of surviving high temperatures and transmitting through heat-treated dairy products. The most common sources of this bacterium are milk powder, and the infant formula industry (Rahimi et al. 2013; Stoeckel et al. 2013; Cetin-Karaca and Morgan 2018). *B. cereus* is a predominant microorganism, it belongs to *B. cereus* sensu lato (s.l.) or known as *B. cereus* group with other genetically linked species as *B. anthracis*, *B. mycoides*, *B. pseudomycoides*, *B. thuringiensis*, *B. cytotoxicus*, *B*. *toyonensis*, and *B. weihenstephanensis* (Sánchez-Chica et al. 2020).

The Gram-positive *B. cereus* organism is responsible for causing diarrhea, emesis, fatal meningitis, tissue destruction, and possibly results in a fatal consequence after contaminated food consumption (Messelhäusser et al. 2007; Frenzel et al. 2015). The reasons for these complications are protein complexes of hemolysin BL (*hbl*) and non-hemolytic enterotoxin (*nhe*) genes. Other enterotoxins consist of a single protein encoded for *bceT* (*B. cereus* enterotoxin), *cytK*, as well as one emetic toxin (*ces*) (Lund et al. 2000; Ehling-Schulz et al. 2006; Hwang and Park 2015).

Multiple antibiotic resistance of *B. cereus* strains is considered one of its riskiness factors in the treatment of infections as they admitted to being widely resistant to various antimicrobials such as β-lactamase, tetracycline, and quinolones (Godic Torkar and Seme 2009; Ranjbar & Shahreza 2017).

The study purposed to investigate the prevalence of *B. cereus*, linked species, virulence factors, evaluation of their antibiotic susceptibility, and product acceptability referenced to the Egyptian standards.

## Materials and methods

### Collection of samples

The samples of 50 whole milk powder, 30 skim milk powder, 30 powdered infant milk formula (for infants from birth till 6 months), and 30 dried milk–cereal-based infant formula (complementary food from 6 month age) were collected from various shops, supermarkets, and pharmacies in Cairo and Giza Governorate, Egypt. These products were imported and repacked in Egyptian factories.

### Enumeration and identification *B. cereus* s.l*.* based on Guinebretiere et al. (2013) and Bennett et al. (2015)

Twenty-five grams of samples were diluted with 225 ml of 0.1% peptone water (Oxoid, CM0009B) to prepare serial dilutions and mixed in a stomacher (Seward^®^400) until complete homogenization. Then, 1 ml of first dilution was distributed and spread on four plates of Mannitol Egg Yolk-Polymyxin agar (MYP) (Oxoid, CM0929) as follows: 0.3 ml, 0.3 ml, 0.3 ml, and 0.1 ml. All plates were incubated at 32 °C for 24 h. Five presumptive positive colonies had been selected for confirmation. For all isolates, different biochemical tests have been applied as Gram stain, catalase, nitrate reduction, Voges–Proskauer, anaerobic fermentation of glucose, tyrosine decomposition, and lysozyme resistance to confirm which of them belonged to the *B. cereus* group and other tests to differentiate species of *B. cereus* s.l., besides evaluation of virulence factors of *B. cereus* strains as follows: motility test, rhizoid growth, hemolytic activity, growth at 6 ℃ and 50 ℃, crystal protein staining, and starch hydrolysis test. *B.* *cereus* ATCC 14,579 and *B. cereus* ATCC 11,778 were used as reference strains for the biochemical and molecular tests. After biochemical identification, the count of *B. cereus* was calculated.

### Molecular identification of B. cereus isolates with detection of its toxigenic genes

#### Extraction of genomic DNA

Genomic DNA was extracted from the culture positively identified as *B. cereus* using Gene JET genomic DNA purification kit (Thermo Fisher, K0721). The supernatant contains DNA stored at − 20 °C.

### PCR for detection of virulence genes

Isolates were tested for *gyrB* gene by primer pair BC1/BC2r and identified as *B. cereus* using positive control (ATCC^®^ 14,579^™^) (Yamada et al. 1999). Besides, testing was performed for the presence of enterotoxigenic genes (*nhe*, *hbl*, *cytK*, and *ces*) using multiplex PCR according to protocol mentioned by Ehling-Schluz et al. (2006). PCR technique for detection of *bceT* gene was referenced to Agata et al. (1995). Primer sets’ PCR amplification, details of its sequences, their specific targets, and amplicon sizes were exhibited in Table S1. The amplification cycles were carried out in aPT-100 Thermocycler (MJ Research, USA). PCR amplification products were analyzed and visualized in 1.5% TBE (Tris Borate EDTA) agarose gels under UV light and all PCR experiments were performed twice for each isolate.

### Measuring and evaluating antibiotic resistance of B. cereus strains

These were carried out using the protocol of the Kirby–Bauer disk diffusion susceptibility method according to Hudzicki (2009). Fresh Five isolate colonies were picked up and suspended in 2 ml of sterile saline, mixed, and incubated at 37 °C. Then, the turbidity of suspension was adjusted by comparing it with the 0.5 McFarland standard solution. A dipped swab (HiMedia, PW009) from an inoculum tube had used for streaking three times on Muller Hinton agar (MH) (Oxoid, CM0337). The antimicrobial disks were placed and dispensed on the surface of the MH agar using sterile forceps. Finally, the inhibition zone had measured after incubation at 35 °C for 18 h; since interpretive guidelines for *B. cereus* susceptibility testing are presently not obtainable, the degree of susceptibility of isolates was determined by following the interpretive guidelines for *Staphylococcus* and other Gram-positive species according to (CLSI 2010; Frenzel et al. 2015). The antimicrobials’ susceptibility disks had used: colistin sulfate, gentamycin, neomycin, tobramycin, and streptomycin (10 μg for each), cefoxitin, cephalothin, chloramphenicol, nalidixic acid, tetracycline and vancomycin (30 μg for each), erythromycin (15 μg), trimethoprim–sulfamethoxazole (25 μg), oxacillin (5 μg) and penicillin (10 U).

### Statistical analysis

Results were analyzed statistically by one-way ANOVA and Chi-square independence test using Microsoft Excel 365 enterprise.

## Results and discussion

The count of *B. cereus* was calculated after enumeration and identification of all isolates. Minimum to maximum values of *B. cereus* in dried samples were 10–8.30 × 10^2^, 10–0.40 × 10^2^, 10–0.30 × 10^2^, and 10–0.80 × 10^2^ CFU/g in 64.0% of whole milk powder, 43.3% skim milk powder, 26.7% powdered infant milk formula and 36.7% milk–cereal-based infant formula, respectively. Based on the mean count of *B. cereus*, there was a significant difference of *(P* < 0.05) between whole milk powder and skim milk powder samples, as well as samples of whole milk powder and powdered infant milk formula (Table 1).

**Table 1:** Statistical analytical results of count of *B. cereus* in the examined samples (CFU/g)

Sample type	Positive samples		Min	Max	Mean ± S.E.M
	No	%			
Whole milk powder (*n* = 50)	32	64.0	10	8.30 × 10^2^	0.57 × 10^2^ ± 0.182 × 10^2a^
Skim milk powder (*n* = 30)	13	43.3	10	0.40 × 10^2^	0.15 × 10^2^ ± 0.027 × 10^2b^
Powdered infant milk formula (*n* = 30)	8	26.7	10	0.30 × 10^2^	0.21 × 10^2^ ± 0.035 × 10^2c^
Milk–cereal-based infant formula (*n* = 30)	11	36.7	10	0.80 × 10^2^	0.32 × 10^2^ ± 0.072 × 10^2d^

*n* number of examined samples; *No.* number of positive samples; *Min.* minimum; *Max.* maximum; *S.E.M.* standard error mean

a, b and a, c superscript between rows indicates significant difference *P* < 0.05. a,d; b,c; c,d and b,d superscript between rows indicates non-significant difference *P* > 0.05

These data were closely similar to results obtained by Rahimi et al. (2013), who proved contamination of examined infant cereal-based formula with *B. cereus* and contributes to the great use of infant food additives or due to the addition of wheat and rice that are rich in starch (Rahimi et al. 2013). Our results were closely related to Aman et al. (2016), who reported *B. cereus* minimum to a maximum count of 10–9 × 10^2^ CFU/g in 19% of examined infant milk powder. Dried milk products have been notified to be contaminated by high concentrations of *B. cereus* vegetative cells, and spores include powdered infant milk formula (Stoeckel et al. 2013; Zhang et al. 2017; Cetin-Karaca and Morgan 2018). Spores of *B. cereus* may enter various dairy products through raw milk (as the soil is the significant source on the farm), and biofilms formed with spores germinate and attach to plant equipment (such as stainless steel) with even resistance to sanitation. Using raw materials of low spore count and improving routine examination to understand the master step during processing at which contamination with spores happened are confirmed to be effective as control and preventive approaches (Stoeckel et al. 2013; Miller et al. 2015; Harada and Nascimento 2021).

**Fig. 1:**
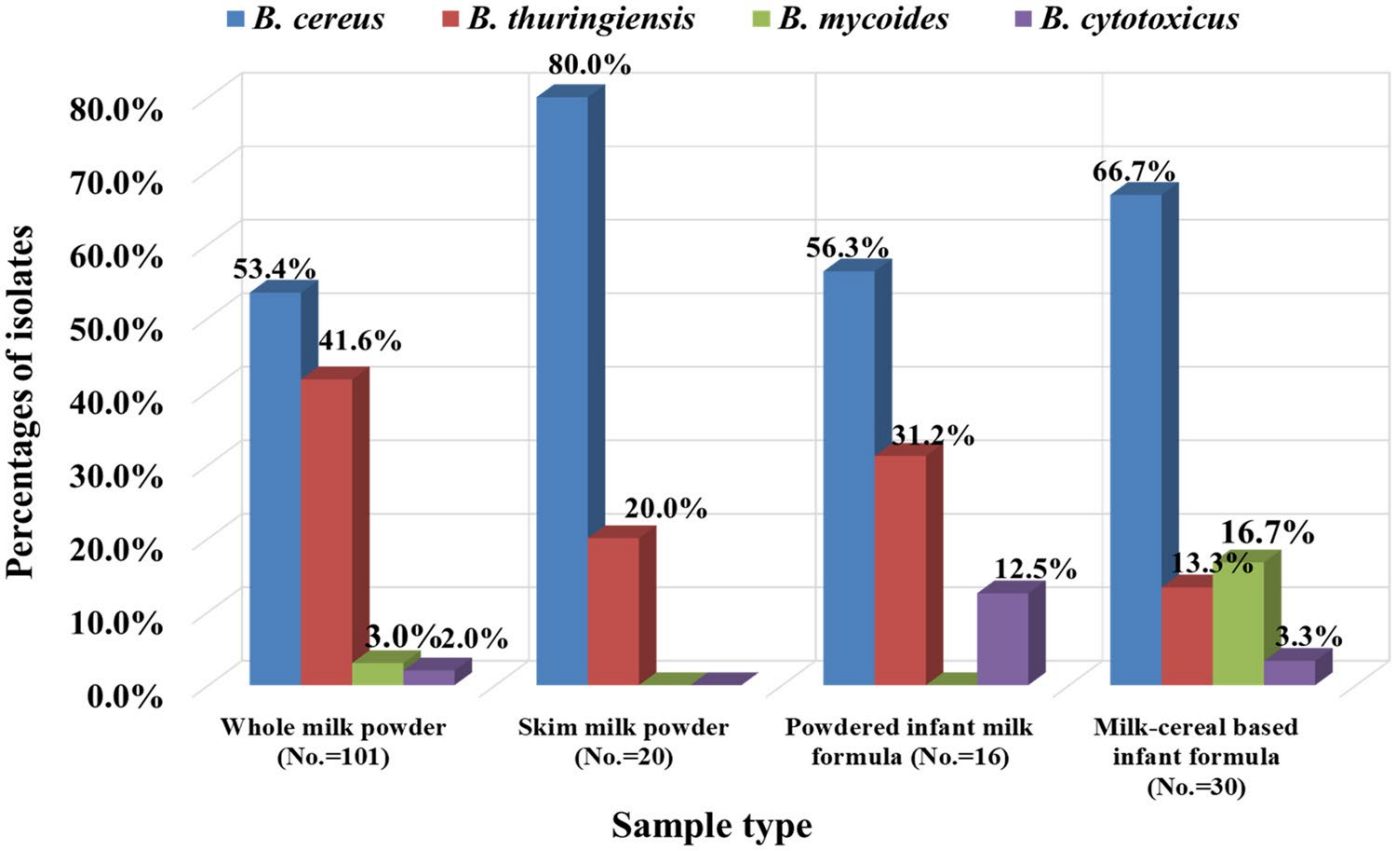
Distribution and differentiation of *B. cereus* group in the examined powdered milk sample. *No.* number of the examined isolates

As presented in Fig. 1, all species have shown lecithinase zone, while *B. cereus* and *B. thuringiensis* were characteristic by hemolytic activity and motility. *B. thuringiensis* was distinguished by crystal toxin protein formation, while *B. mycoides* was characterized by rhizoid growth on nutrient agar. However, *B. cytotoxicus* was identified by the ability to grow at 50 °C, as other members were not able to grow at this temperature. A total of 167 isolates of the *B. cereus* group were identified as follows: 101 for whole milk powder (53.4% *B. cereus*, 41.6% *B. thuringiensis*, 3.0% *B. mycoides*, and 2.0% *B. cytotoxicus)*, 20 for skim milk powder (80.0% *B. cereus* and 20.0% *B. thuringiensis*), 16 for powdered infant milk formula (56.3% *B. cereus*, 31.2% *B. thuringiensis*, and 12.5% *B. cytotoxicus*) and 30 for milk–cereal-based infant formula (66.7% *B. cereus*, 13.3% *B. thuringiensis*, 16.7% *B. mycoides* and 3.3% *B. cytotoxicus*). Furthermore, Hwang and Park 2015 recognized 41.8% *B. cereus* and 58.2% *B. thuringiensis* from 99 powdered infant formula samples.

*B. thuringiensis* is a common pathogen in milk; it has been stated to produce enterotoxins in food and exhibit cytotoxicity (Johler et al. 2018). However, outbreaks associated with this organism had been discussed in a recent report by EFSA (2016), which declared the insistent demand to further studies to develop a risk assessment of *B. thuringiensis* in food poisoning outbreaks. While thermo-tolerant *B. cytotoxicus* has carried the cytotoxin K gene. Besides, it was isolated from milk-based foods for 179 infants that described the possibility of causing outbreaks of food poisoning, which results in 180 considered *B. cytotoxicus* as a risk factor, especially for neonates (Guinebretiere et al. 2013; Zhang et al. 2017). This species is linked to three deaths of infants in France due to causing necrotic enteritis (Lequin et al. 2005). In addition, infant’s infection with *B. cereus* had highly increased recently as announced by Frenzel et al. (2015), so EFSA 2016 recommended that this pathogen and its spores in dried milk infant formula must be at least as possible (< 100 CFU/g).

It is highly known that spores of these bacteria after powder milk reconstitution can vegetate, especially with using un-cleaned water or poor equipment sanitization. These unhygienic conditions may result in toxin production in the time of household powder preparation, handling, and during retaining of baby bottles. In addition, polluted ingredients added after drying may cause recontamination or surroundings from drying till packaging (Stoeckel et al. 2013; Cetin-Karaca and Morgan 2018).

*B. cereus* can produce sphingomyelinase and lecithinase that destroy the membrane of the cell body. They have a hemolytic activity which plays a synergistic role in dissolving red blood cells (RBCs) which also have been associated with multiple outbreaks (Hwang and Park 2015). Consequently, specific tests had performed to determine their capability to express hemolysis and hydrolysis of starch. All isolates had lecithinase activity, and 89.9% of *B. cereus* isolates were strongly hemolytic. Isolates identified from milk–cereal-based infant formula had the highest percentage of showing hemolysis (95.0%), nearly like data recorded by Sadek et al. (2018). A powerful tool in the pathogenicity of *B. cereus* is the hydrolysis of starch, which had occurred in 68.7% of isolates. All *B. cereus* isolates belonging to milk–cereal-based infant formula were productive to starch hydrolysis (Table 2). Hwang and Park 2015 detected the ability of *B. cereus* to hydrolyze starch in 35.0% of infant formula isolates.

**Table 2:** Pattern of hemolytic and starch hydrolysis activities among *B. cereus* isolates obtained from the examined samples

Type of samples	Hemolytic activity of *B. cereus* isolates				Starch hydrolysis of *B. cereus* isolates			
	Strong		Weak		Hydrolytic		Non-hydrolytic	
	No	%	No	%	No	%	No	%
Whole milk powder (No. = 54)	49	90.7	5	9.3	35	64.8	19	35.2
Skim milk powder (No. = 16)	13	81.2	3	18.8	6	37.5	10	62.5
Powdered infant milk formula (No. = 9)	8	88.9	1	11.1	7	77.8	2	22.2
Milk–cereal-based infant formula (No. = 20)	19	95.0	1	5.0	20	100.0	0	0.0
Total (No. = 99)	89	89.9	10	10.1	68	68.7	31	31.3

*No.* number of the examined isolates

As exhibited in Table 3, a total number of 99 *B. cereus* isolates were positive for *gyrB* and confirmed to carry the *B. cereus* gene as outlined in Fig. S1. Only two isolates recovered from milk–cereal-based infant formula harbored *hbl* gene, these data approached to finding notified by Sadek et al. (2018). The most excessively distributed gene was *nhe* in a total percentage of 77.8 as 100.0% for both skim milk powder and powdered infant milk formula. Our results revealed that *hbl* gene was less prevalent than *nhe* gene as also demonstrated in previous studies (Hwang and Park 2015; Sadek et al. 2018). Another toxigenic gene is *cytK*, which is represented by 72.7% of the total examined isolates as 47 of them belonged to whole milk powder samples in a percentage of 87.0. This is the only product that harbored the emetic cereulide toxin gene in a percent (29.6%) by 16 isolates. The cytotoxic gene has hemolysis and necrosis activities on cells and is responsible for causing fatal poisoning outbreaks (Hwang and Park 2015). The cereulide toxin may lead to the severe consequence of damage to the liver, multiorgan dysfunction, and a link to diabetes (Frenzel et al. 2015; EFSA 2016).

**Table 3:** Prevalence of virulence enterotoxigenic genes of *B. cereus* isolated from the examined powdered milk samples

Sample type	Confirmed *B. cereus* *gyrB*	Virulence enterotoxigenic genes							
		*nhe*	*hbl*	*cytK*	*ces*	*bceT*	*nhe*, *cytK*, *bceT*	*nhe*, *cytK*, *ces*, *bceT*	*nhe*, *cytK hbl*, *bceT*
Whole milk powder (No. = 54)	54 (100.0%)	41^a^ (75.9%)	0^a^	47^a^ (87.0%)	16^b^ (29.6%)	31^a^ (57.4%)	22 (40.7%)	3 (5.6%)	0
Skim milk powder (No. = 16)	16 (100.0%)	16^d^ (100.0%)	0^a^	12^a^ (75.0%)	0^a^	14^b^ (87.5%)	10 (62.5%)	0	0
Powdered infant milk formula (No. = 9)	9 (100.0%)	9^a^ (100.0%)	0^a^	3^b^ (33.3%)	0^d^	7^d^ (77.8%)	3 (33.3%)	0	0
Milk–cereal-based infant formula (No. = 20)	20 (100.0%)	11^e^ (55.0%)	2^a^ (10.0%)	10^c^ (50.0%)	0^a^	15^d^ (75.0%)	6 (30.0%)	0	2 (10.0%)
Total	99 (100.0%)	77 (77.8%)	2 (2.0%)	72 (72.7%)	16 (16.2%)	67 (67.7%)	41 (41.4%)	3 (3.0%)	2 (2.0%)

*No.* number of the examined isolates

a,b; a,c; b,c and d,e in the same column indicate significant difference *P* < 0.05

a,a; a,d; a,e; b,d and d,d in the same column indicate non-significant difference *P* > 0.05

As displayed in Figs. S2 and S3, *bceT* gene that is one of diarrheal enterotoxins was detected in a total percentage of 67.7 of examined isolates and distributed among all products. A total of 41 isolates expressed the 3 toxigenic genes (*nhe*, *cytK* and *bceT*) in the following manner: 22 (40.7%), 10 (62.5%), 3 (33.3%), and 6 (30.0%) isolates from whole milk powder, skim milk powder, powdered infant milk formula, and milk–cereal-based infant formula, respectively, while uniquely 3 (5.6%) from whole milk powder isolates harboring 4 genes (*nhe*, *cytK*, *ces*, and *bceT*) and 2 (10.0%) from dried milk–cereal-based infant formula were expressed (*nhe*, *cytK*, *hbl*, and *bceT*). Based on the prevalence of *nhe* and *cytK* genes, there was a significant difference (*P* < 0.05) between *B. cereus* strains isolated from skim milk powder and milk–cereal-based infant formula. In addition, there was a significance difference between *B. cereus* strains of whole and skim milk powder for *ces* and *bceT* genes, as well as between whole milk powder and milk–cereal-based infant formula for *ces* and *cytK* gene.

While based on the prevalence of *cytK* and *bceT* genes, there was a significant difference between *B. cereus* strains isolated from powdered infant milk formula and milk–cereal-based infant formula. Besides a significant difference between *B. cereus* strains on harboring *cytK* gene between whole milk powders and powdered infant milk formula, no significant difference was found (*P* > 0.05) in carrying *hbl* gene for all isolated strains as shown in Table 3. In this study, *B. cereus* strains were found to harbor more than toxigenic genes such as nhe, *bceT*, and/or *cytK*, so they may have the possibility to result in emetic and diarrheal food poisoning concurrently. Our findings were almost like the research reported by Rahimi et al. (2013), who concluded that 6.7% of *B. cereus* isolates from dried baby food with milk-based harbored *nhe*, *hbl*, and *bceT* genes.

However, Di pinto et al. (2013) reported comparable to our data, a total of 12 *B. cereus* strains were isolated from five powdered infant milk formula samples that harbored a minimum one from the following genes: (*cytK*, *hbl*, and nhe). While Sadek et al. (2018) had revealed the ability of their isolated *B. cereus* strains from milk-based baby formula to carry enterotoxigenic genes in the following proportions: 95.5% (43) for *cytK* gene, 71.1% (32) for *nhe*, and 11.1% (5) for *hbl* genes.

The *hbl*, *nhe*, and *cytK* toxigenic genes have caused food poisoning in individuals, as *hbl* and *nhe* are responsible for hemolytic and cytotoxic properties, while *cytK* has been recorded to cause diarrhea with blood (Hwang and Park 2015).

**Fig. 2:**
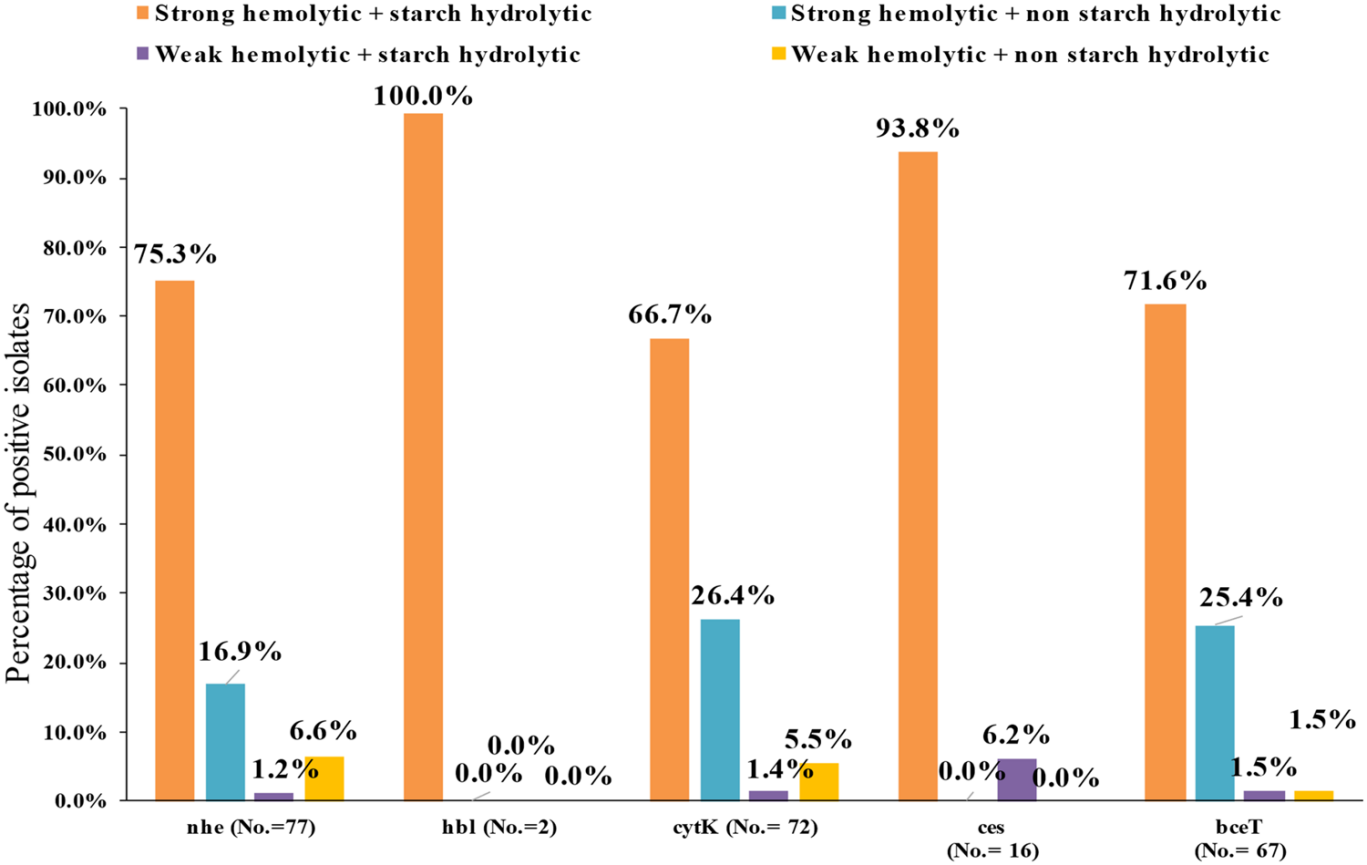
Relationship of hemolytic activity, starch hydrolysis and harboring genes *nhe*, *hbl*, *cytK*, *ces*, and *bceT* of *B. cereus* strains isolated from dried milk and infant formulas. *No.* number of the *B. cereus* strains

In Fig. 2, strains of *B. cereus* that carried *nhe*, *hbl*, *cytK*, *ces*, and *bceT* were able to show strong hemolysis with starch hydrolysis in a percent of 75.3%, 100.0%, 66.7%, 93.8%, and 71.6%, respectively. These proved the high relation between harboring toxigenic genes and the exhibition of virulence features (lecithinase, strong hemolysis, and starch hydrolysis).

Although some toxigenic strains were weakly hemolytic and could hydrolyze starch, they harbored *nhe*, *cytK*, *ces*, and *bceT* genes, in percent of 1.2, 1.4, 6.2, and 1.5, respectively. Although some strains were not hydrolyzed starch and exhibited weak hemolysis on blood agar, they could express enterotoxigenic genes in the low percent, 6.6% *nhe*, 5.5% *cytK*, and 1.5% *bceT* gene, while Organji et al. (2015) informed that all *B. cereus* strains obtained from infant formula milk displayed strong hemolytic character and fewer tendencies to express *cytK* and *hbl*.

Pirhonen et al. (2005) announced that *B. cereus* strains that showed extremely strong hemolysis are the reason for food poisoning. As reported by Andersson et al. (2004), 27.0% of isolated strong hemolytic *B. cereus* had not produced emetic toxin, while the other 77 isolates demonstrated weak hemolysis with the production of *ces* toxin.

We assumed a complete association (100.0%) between expressing *hbl* gene, hemolytic, and starch hydrolytic (Fig. 2). Some researchers announced that the test of starch hydrolysis is an indicator for *B. cereus* emetic isolates (Ehling-Schulz et al. 2005; Pirhonen et al. 2005), while Hwang and Park (2015) concluded an intense relationship between the ability of *B. cereus* to hydrolyze starch and the expression of *hbl* and *cytK* genes.

In measuring antibiotic sensitivity of the isolated toxigenic *B. cereus* strains, these belonged to skim milk powder that had shown the most similarity in their pattern and followed by whole milk powder strains. All skim and whole milk powder strains were inhibited by chloramphenicol, gentamycin, nalidixic acid, tetracycline, tobramycin, streptomycin, and vancomycin, and it resisted the cefoxitin, cephalothin, colistin sulfate, neomycin, trimethoprim–sulfamethoxazole, oxacillin, and penicillin (Table 4).

**Table 4:** Antibiotics susceptibility evaluation of *B. cereus* strains

Product Antibiotic	Whole milk powder (No. = 54)			Skim milk powder (No. = 16)			Powdered infant milk formula (No. = 9)			Milk–cereal-based infant formula (No. = 20)		
	*R*	*I*	*S*	*R*	*I*	*S*	*R*	*I*	*S*	*R*	*I*	*S*
Cefoxitin	54^a^ 100%	0	0	16^a^ 100%	0	0	9^a^ 100%	0	0	20^a^ 100%	0	0
Cephalothin	54^a^ 100%	0	0	16^ab^ 100%	0	0	7^b^ 77.8%	0	2 22.2%	20^ab^ 100%	0	0
Chloramphenicol	0^a^	0	54 100%	0^a^	0	16 100%	8^b^ 88.9%	1 11.1%	0	0^a^	0	20 100%
Colistin sulfate	54^a^ 100%	0	0	16^a^ 100%	0	0	9^a^ 100%	0	0	20^a^ 100%	0	0
Erythromycin	16^a^ 29.6%	22 40.7%	16 29.6%	0^b^	16 100%	0	0^ab^	9 100%	0	5^ab^ 25%	0	15 75%
Gentamycin	0^a^	0	54 100%	0^a^	0	16 100%	0^a^	0	9 100%	0^a^	0	20 100%
Nalidixic acid	0^a^	0	54 100%	0^ab^	0	16 100%	2^b^ 22.2%	2 22.2%	5 55.6%	9^c^ 45%	5 25%	6 30%
Neomycin	54^a^ 100%	0	0	16^a^ 100%	0	0	9^a^ 100%	0	0	20^a^ 100%	0	0
Tetracycline	0^a^	0	54 100%	0^a^	0	16 100%	8^b^ 88.9%	0	1 11.1%	3^b^ 15%	0	17 85%
Tobramycin	0^a^	0	54 100%	0^a^	0	16 100%	0^a^	0	9 100%	0^a^	0	20 100%
Trimethoprim–sulfamethoxazole	54^a^ 100%	0	0	16^a^ 100%	0	0	9^a^ 100%	0	0	20^a^ 100%	0	0
Oxacillin	54^a^ 100%	0	0	16^a^ 100%	0	0	9^a^ 100%	0	0	20^a^ 100%	0	0
Penicillin	54^a^ 100%	0	0	16^a^ 100%	0	0	9^a^ 100%	0	0	20a 100%	0	0
Streptomycin	0^a^	0	54 100%	0^a^	0	16 100%	0^a^	0	9 100%	0^a^	0	20 100%
Vancomycin	0^a^	0	54 100%	0^a^	0	16 100%	0^a^	0	9 100%	0^a^	0	20 100%

*No.* number of the examined isolates, *R* resistant, *I* intermediate resistant, *S* susceptible

a,b; b,b; a,c; ab,c in the same row indicate significant difference *P* > 0.05

ab,b; ab,a; ab,ab; b,c in the same row indicate non-significant difference *P* < 0.05

Hundred percent among strains identified from our examined infant foods had resisted cefoxitin, colistin sulfate, neomycin, trimethoprim–sulfamethoxazole, oxacillin, and penicillin antibiotics, while they were susceptible to gentamycin, tobramycin, streptomycin, and vancomycin in a proportion of 100.0%. For remaining antibiotics, these strains had shown different liability to antibiotics in the following manner: 77.8% and 100.0% were resistant to cephalothin for powdered infant milk formula and milk–cereal-based infant formula, respectively, and 88.9% and 15.0% for tetracycline. Exclusively, powdered infant milk formula strains had grown well in the occurrence of chloramphenicol with a percentage of 88.9 and 22.2 for nalidixic acid. Finally, five (25.0%) strains from milk–cereal-based infant formula were resistant to erythromycin and nine (45.0%) to nalidixic acid (Table 4).

Osama et al. (2020) announced that *B. cereus* isolated from Egyptian dairy products were 100% resistant to colistin, 67.9% resistant to streptomycin, 2.6% resistant to tetracycline, and 5.6% resistant to erythromycin. However, *B. cereus* strains identified by Kim et al. (2015) showed susceptibility to vancomycin, gentamicin, and tetracycline but impedance to β‐lactam antibiotics such as penicillin and oxacillin.

Ranjbar and Shahreza (2017) presented that resistance of *B. cereus* from nine milk-based baby food samples that was in a percent of 100, 77.7, 66.6, 44.4, and 11.1 to penicillin, tetracycline, oxacillin, trimethoprim-sulfamethoxazole, and chloramphenicol, respectively.

As presented in Table 4, the resistance of *B. cereus* strains obtained from powdered infant milk formula to cephalothin and nalidixic acid were significantly different (*P* < 0.05) with whole milk powder strains. While based on resistance to chloramphenicol and tetracycline, strains from powdered infant milk formula were significantly different (*P* < 0.05) from whole milk powder, skim milk powder, and milk–cereal-based infant formula strains. As well as based on the results of *B. cereus* strains’ resistance to erythromycin, there was a significant difference between whole and skim milk powder strains. In addition, there was a significant difference between *B. cereus* strains isolated from whole milk powder and milk–cereal-based infant formula referred to their resistance to nalidixic acid and tetracycline antibiotics, as well as between skim milk powder and milk–cereal-based infant formula.

With a comparison of *B. cereus* strains based on antibiotics resistance, strains obtained from powdered infant milk formula and whole milk powder exhibited a higher prevalence statistically significant difference (*P* < 0.05) than isolated strains from skim milk powder and milk–cereal-based infant formula. There was no significant difference (*P* > 0.05) in antimicrobial resistance of all obtained *B. cereus* strains toward cefoxitin, colistin sulfate, gentamycin, neomycin, tobramycin, trimethoprim–sulfamethoxazole, oxacillin, penicillin, streptomycin, and vancomycin.

Several reasons were for expressing drug resistance as the variation of the strain’s origin, transferring of antibiotic resistance, and misusing in treatments. Therefore, it is significant to study the manner of antimicrobial resistance of *B. cereus* isolated from dairy food with the more restricted policy in the utilization of antimicrobials (Ranjbar and Shahreza 2017; Osama et al. 2020). As deduced from previous results, the *B. cereus* strains showed resistance to more than one type of antibiotic, so suggested more attention to effective antibiotic therapy to eradicate *B. cereus* infections. As well as they displayed a significant statistical variation in harboring virulence genes and resistance to antimicrobials, which may be contributing to the fact of having a plastic genome that characterized *B. cereus* by horizontal gene transmit and results in genetic diversity as reported by Osman et al. (2018).

**Fig. 3:**
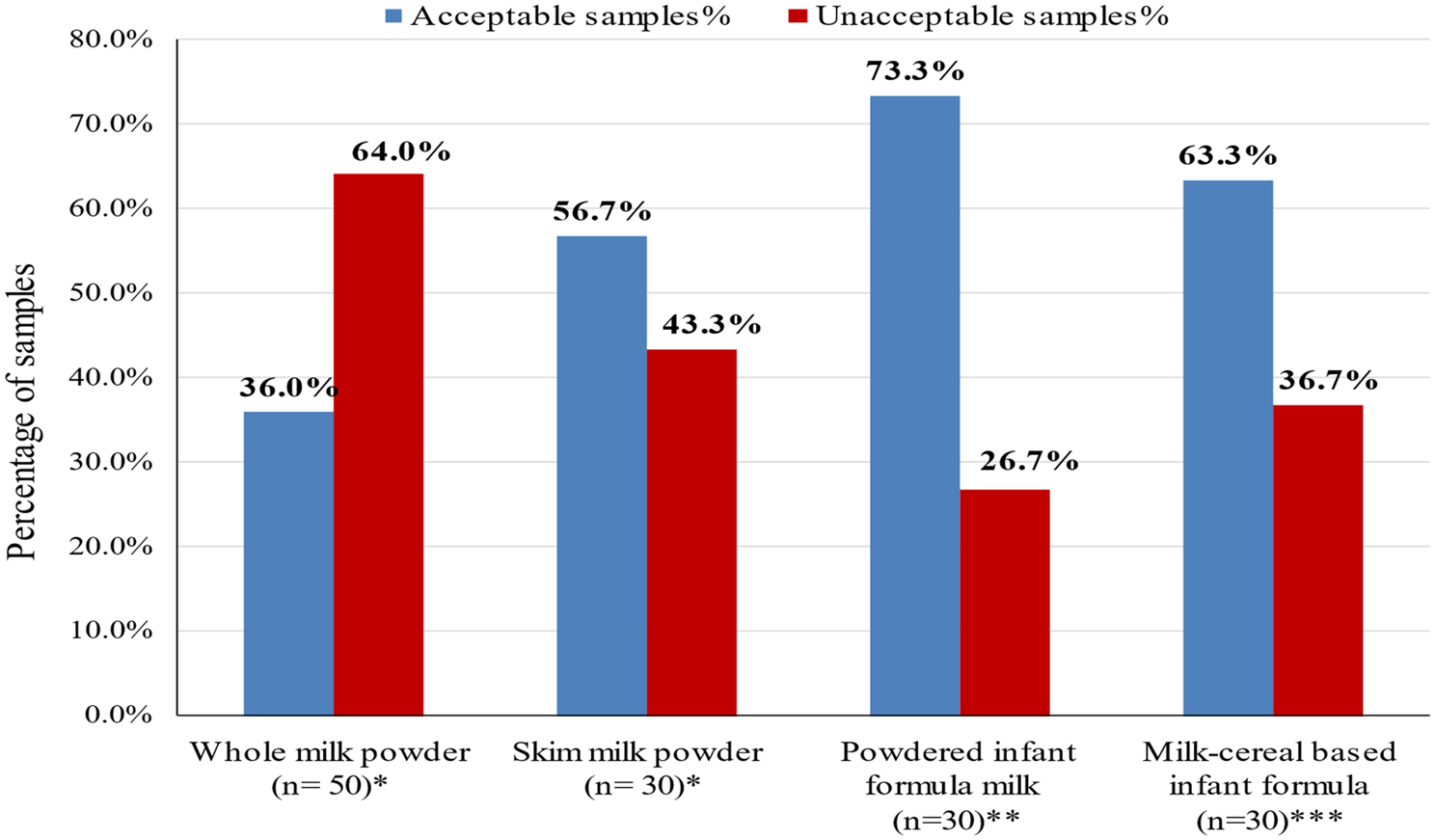
Percentages of acceptability degree of the examined samples depend on Egyptian standards based on *B. cereus* count. *n* number of the examined samples; *Egyptian standards (ES: 1780/ 2014); **Egyptian standards (ES: 2072/2006); ***Egyptian standards (ES: 3284/2005)

Referred to Egyptian standards (2006, 2014), the milk powders and infant formula shall be free from pathogenic microorganisms, so samples found to contain *B. cereus* are considered unacceptable. However, Egyptian standards (2005) announced milk–cereal-based infant formula is acceptable without *B. cereus*. Consequently, our samples were satisfactory and fit for consumption in the following percentages: 36, 56.7, 73.3, and 63.3 for whole, skim milk powders, powdered infant milk formula, and milk–cereal-based infant formula, respectively, shown in Fig. 3. European Commission (2005) amended a legal limit of *B. cereus* count of < 50 CFU/g, for powder infant milk formula intended from birth till < 6 month age. Food Standards Australia New Zealand (FSANZ 2004) pronounced *B. cereus* might reach its infectious dose within 4 h when stored at room temperature with a primary count of 10^2^ CFU/g. One of the products that have a high risk is whole milk powder, especially when reconstituted with cold water as mentioned on its labels, and it mainly depends on the time between preparation and consumption. As documented by EFSA 2016, cells or even spores of *B. cereus* in a count of > 10^4^ CFU/g will produce diarrheal toxins in the human gut and intestine. Timely manufacturing practice and actualizing food safety management systems are needed for supreme safe production (Ibrahim et al. 2021).

## Conclusion

This study shows that *B. cereus* harbors several toxigenic genes that could contaminate dried milk products, particularly milk formula for pediatrics; this pathogen poses a possible food safety risk. Special attention to the progress of *B. cereus* antibiotic resistance is required for effective treatment and early recovery. The variation in identified virulence genes and antibiotic resistance between *B. cereus* strains from different examined samples proved that the type of product was relevant to the count and toxigenicity of *B. cereus* strains. Consumers should confirm good practices such as proper holding times and storage temperatures. More limitations by Egyptian and international authorities should be applied to control and prevent *B. cereus* contamination in dried milk for saving low immune system consumers from multiple health problems. Finally, dried milk and powdered infant milk formula should be checked, monitored recurrently, and the application of food safety management systems.

## Supplementary Information

Below is the link to the electronic supplementary material.

Supplementary file1 (DOCX 3172 kb)

## Data Availability

The data generated or analyzed during this study are included in this published article and its supplementary information files.

## References

[CR1] Agata N, Ohta M, Arakawa Y, Mori M (1995). The bceT gene of *Bacillus cereus* encodes an enterotoxic protein. Microbiology.

[CR2] Aman IM, Abbas EM, Elkassas WM (2016). Safety of infant milk powder sold at Kafrelsheikh governorate markets, Egypt. Int J Innov Res Sci Engin.

[CR3] Andersson MA, Jääskeläinen EL, Shaheen R, Pirhonen T, Wijnands LM, Salkinoja-Salonen MS (2004). Sperm bioassay for rapid detection of cereulide-producing *Bacillus cereus* in food and related environments. Int J Food Microbiol.

[CR4] Bennett RW, Tallent SM, Hait JM (2015) *Bacillus cereus*. In: Salfinger, Y., Tortorello, M.L. (eds) Compendium of Methods for the Microbiological Examination of Foods, 5th edition. American Public Health Association, Washington, DC, USA. Chapter 31, pp. 375–390.

[CR5] Cetin-Karaca H, Morgan MC (2018). Inactivation of *Bacillus cereus* spores in infant formula by combination of high pressure and trans-cinnamaldehyde. LWT.

[CR6] CLSI (2010) Clinical and Laboratory Standards Institute. 2010. Performance standards for antimicrobial susceptibility testing: 20th informational supplement. M100-S20., Wayne, PA.

[CR7] Di Pinto A, Bonerba E, Bozzo G, Ceci E, Terio V, Tantillo G (2013). Occurence of potentially enterotoxigenic *Bacillus cereus* in infant milk powder. Eur Food Res Technol.

[CR8] EFSA (2016). European food safety authority. Risks for public health related to the presence of *Bacillus cereus* and other *Bacillus* spp. including *Bacillus thuringiensis* in foodstuffs. EFSA J.

[CR9] Ehling-Schulz M, Vukov N, Schulz A, Shaheen R, Andersson M, Märtlbauer E, Scherer S (2005). Identification and partial characterization of the nonribosomal peptide synthetase gene responsible for cereulide production in emetic *Bacillus cereus*. Appl Environ Microbiol.

[CR10] Ehling-Schulz M, Guinebretiere MH, Monthán A, Berge O, Fricker M, Svensson B (2006). Toxin gene profiling of enterotoxic and emetic *Bacillus cereus*. FEMS Microbiol Lett.

[CR11] Egyptian Standards (2005) Processed Cereal-based for foods for Infants and Children. Egyptian Organization for Standardization and Quality Control, Ministry of Industry (ES: 3284/2005)

[CR12] Egyptian Standards (2006) Infant formula. Egyptian Organization for Standardization and Quality Control, Ministry of Industry (ES: 2072/2006).

[CR13] Egyptian Standards (2014) Milk powder and cream powder. Egyptian Organization for Standardization and Quality Control, Ministry of Industry (ES: 1780/2014).

[CR14] European Commission (2005). Commission regulation (EC) No 2073/2005. Official J Eur Union.

[CR15] Frenzel E, Kranzler M, Stark TD, Hofmann T, Ehling-Schulz M (2015). The endospore-forming pathogen *Bacillus cereus* exploits a small colony variant-based diversification strategy in response to aminoglycoside exposure. Mbio.

[CR16] FSANZ (2004) Food standards Australia New Zealand, *Bacillus cereus* limits in infant formula. Final Assessment Report, Application, A454.

[CR17] Godic Torkar K, Seme K (2009). Antimicrobial susceptibility, beta-lactamase and enterotoxin production in *Bacillus cereus* isolates from clinical and food samples. Folia Microbiol (praha).

[CR18] Guinebretiere MH, Auger S, Galleron N, Contzen M, De Sarrau B, De Buyser ML, Lamberet G, Fagerlund A, Granum PE, Lereclus D, De Vos P, Nguyen-The C, Sorokin A (2013). *Bacillus cytotoxicus* sp. nov. is a novel thermotolerant species of the *Bacillus cereus* group occasionally associated with food poisoning. Int J Syst Evol Microbiol.

[CR19] Harada AMM, Nascimento MS (2021). Effect of dry sanitizing methods on *Bacillus cereus* biofilm. Braz J Microbiol.

[CR20] Hudzicki J (2009). Kirby-Bauer disk diffusion susceptibility test protocol. Am Soc Microbiol.

[CR21] Hwang J, Park J (2015). Characteristics of enterotoxin distribution, hemolysis, lecithinase, and starch hydrolysis of *Bacillus cereus* isolated from infant formulas and ready-to-eat foods. J Dairy Sci.

[CR22] Ibrahim AS, Saad MF, Hafiz NM (2021). Safety and quality aspects of whole and skimmed milk powders. Acta Sci Pol Technol Aliment.

[CR23] Johler S, Kalbhenn EM, Heini N, Brodmann P, Gautsch S, Bağcioğlu M, Contzen M, Stephan R, Ehling-Schulz M (2018). Enterotoxin production of *Bacillus thuringiensis* isolates from biopesticides, foods, and outbreaks. Front Microbiol.

[CR24] Kim CW, Cho SH, Kang SH, Park YB, Yoon MH, Lee JB, No WS, Kim JB (2015). Prevalence, genetic diversity, and antibiotic resistance of *Bacillus cereus* isolated from Korean fermented soybean products. J Food Sci.

[CR25] Lequin MH, Vermeulen JR, Van Elburg RM, Barkhof F, Kornelisse RF, Swarte R, Govaert PP (2005). *Bacillus cereus* meningoencephalitis in preterm infants: neuroimaging characteristics. AJNR Am J Neuroradiol.

[CR26] Lund T, De Buyser M, Granum PE (2000). A new cytotoxin from *Bacillus cereus* that may cause necrotic enteritis. Mol Microbiol.

[CR27] Messelhäusser U, Fricker M, Ehling-Schulz M, Ziegler H, Elmer-Englhard D, Kleih W, Busch U (2007). Real-time PCR system for the detection of *Bacillus cereus* (emetic type) in food. J Consum.

[CR28] Miller RA, Kent DJ, Watterson MJ, Boor KJ, Martin NH, Wiedmann M (2015). Spore populations among bulk tank raw milk and dairy powders are significantly diff erent. J Dairy Sci.

[CR29] Organji SR, Abulreesh HH, Elbanna K, Osman GEH, Khider M (2015). Occurrence and characterization of toxigenic *Bacillus cereus* in food and infant feces. Asian Pac J Trop Biomed.

[CR30] Osama R, Ahmed MFE, Abdulmawjood A, Al-Ashmawy M (2020). Prevalence and antimicrobial resistance of *Bacillus cereus* in milk and dairy products. Mansoura Vet Med J.

[CR31] Osman KM, Kappell AD, Orabi A (2018). Poultry and beef meat as potential seedbeds for antimicrobial resistant enterotoxigenic *Bacillus* species: a materializing epidemiological and potential severe health hazard. Sci Rep.

[CR32] Pirhonen TI, Andersson MA, Jääskeläinen EL, SalkinojaSalonen MS, Honkanen-Burzalski T, Johansson TML (2005). Biochemical and toxic diversity of *Bacillus cereus* in a pasta and meat dish associated with a food-poisoning case. Food Microbiol.

[CR33] Rahimi E, Jalali M, Abdos F, Momtaz H, Baghbadorani ZT (2013). *Bacillus cereus* in infant foods: prevalence study and distribution of enterotoxigenic virulence factors in Isfahan province, Iran. Sci World J.

[CR34] Ranjbar R, Shahreza MHS (2017). Prevalence, antibiotic-resistance properties and enterotoxin gene profile of *Bacillus cereus* strains isolated from milk-based baby foods. Trop J Pharm Res.

[CR35] Sadek ZI, Abdel-Rahman MA, Azab MS, Darwesh OM, Hassan MS (2018). Microbiological evaluation of infant foods quality and molecular detection of *Bacillus cereus* toxins relating genes. Toxicol Rep.

[CR36] Sánchez-Chica J, Correa MM, Aceves-Diez AE, Castañeda-Sandoval LM (2020). Genetic and toxigenic diversity of *Bacillus cereus* group isolated from powdered foods. J Food Sci Technol.

[CR37] Stoeckel M, Westermann A, Atamer Z, Hinrichs J (2013). Thermal inactivation of *Bacillus cereus* spores in infant formula under shear conditions. Dairy Sci Technol.

[CR38] WHO (2007) World Health Organization. Food safety & food-borne illness. Fact Sheet No. 237, World Health Organization, Geneva, Switzerland.

[CR39] Yamada S, Ohashi E, Agata N, Venkateswaran K (1999). Cloning and nucleotide sequence analysis of *gyrB* of *Bacillus cereus*, *B. thuringiensis*, *B. mycoides*, and *B. anthracis* and their applications to the detection of *B. cereus* in rice. Appl Environ Microbiol.

[CR40] Zhang Y, Chen J, Feng C, Zhan L, Zhang J, Li Y, Yang Y, Chen H, Zhang Z, Zhang Y, Mei L, Li H (2017). Quantitative prevalence, phenotypic and genotypic characteristics of *Bacillus cereus* isolated from retail infant foods in China. Foodborne Pathog Dis.

