# Supplementary material for: Prevalence of Bacillus cereus in dairy powders focusing on its toxigenic genes and antimicrobial resistance
Source: Arch Microbiol. 2022 May 19;204(6):339. doi: 10.1007/s00203-022-02945-3 (PMC9120150; doi:10.1007/s00203-022-02945-3)
Supplement: Supplementary file 1 — Supplementary file1 (DOCX 3172 kb) [file 203_2022_2945_MOESM1_ESM.docx]

**Table S1.** Primers sequence for detecting *B. cereus* enterotoxigenic isolates.

| **Target gene** | | **Primer** | **Primer sequence (5′➔3′)** | **Ampliﬁed**  **fragment**  **size (bp)** | **Reference** |
| --- | --- | --- | --- | --- | --- |
| *gyrB* | | BC1F | ATT GGT GAC ACC GAT CAA ACA | 365 | **Yamada et al., 1999** |
|  |  | BC2rR | TCA TAC GTA TGG ATG TTA TTC |  |  |
| **Multiplex PCR** | *nhe* | NA2F | AAGCIGCTCTTCGIATTC | 766 | **Ehling-Schluz et al., 2006** |
|  |  | NB1R | ITIGTTGAAATAAGCTGTGG |  |  |
|  | *hbl* | HD2F | GTAAATTAIGATGAICAATTTC | 1091 |  |
|  |  | HA4R | AGAATAGGCATTCATAGATT |  |  |
|  | *cytK* | F2 | ACA GAT ATC GGI CAA AAT GC | 421 |  |
|  |  | R7 | CAA GTI ACT TGA CCI GTT GC |  |  |
|  | *Ces* | CesF1 | GGTGACACATTATCATATAAGGTG | 1271 |  |
|  |  | CesR2 | GTAAGCGAACCTGTCTGTAACAACA |  |  |
| *bceT* | | ETF | TTACATTACCAGGACGTGCTT | 428 | **Agata et al., 1995** |
|  |  | ETR | TGTTTGTGATTGTAATTCAGG |  |  |


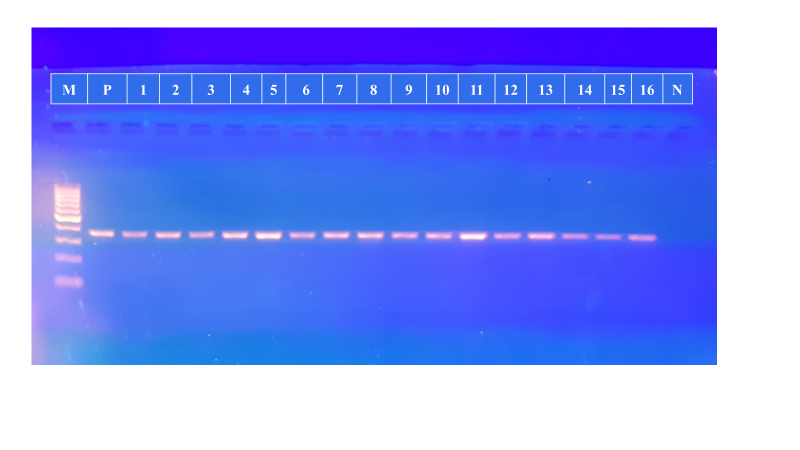
**Fig. S1.** Agarose gel of PCR used for detection of *B. cereus* target *gyrB* gene (365bp).

M: molecular size marker (100bp DNA ladder), P*:* Positive control of *Bacillus cereus* (ATCC® 14579^TM^)*,* 1-16: Representative positive isolates, N: Negative control.


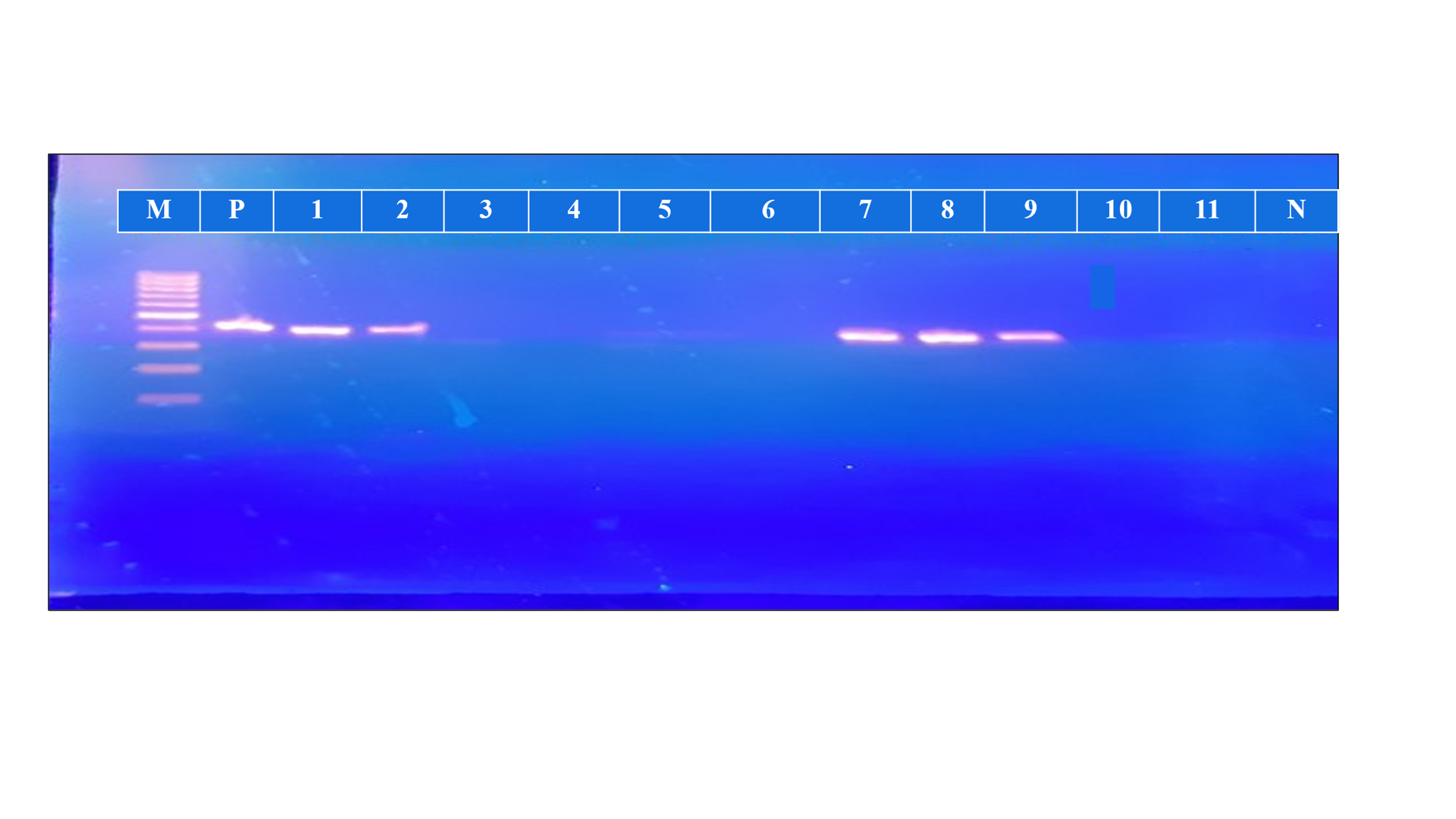
**Fig. S2.** Image of PCR products of enterotoxigenic *bceT* gene (428bp).

M: molecular size marker (100bp DNA ladder), P*:* Positive control of *Bacillus cereus* (ATCC® 14579^TM^), Lines of 1, 2, 7-9: Representative positive strains, N: Negative control.


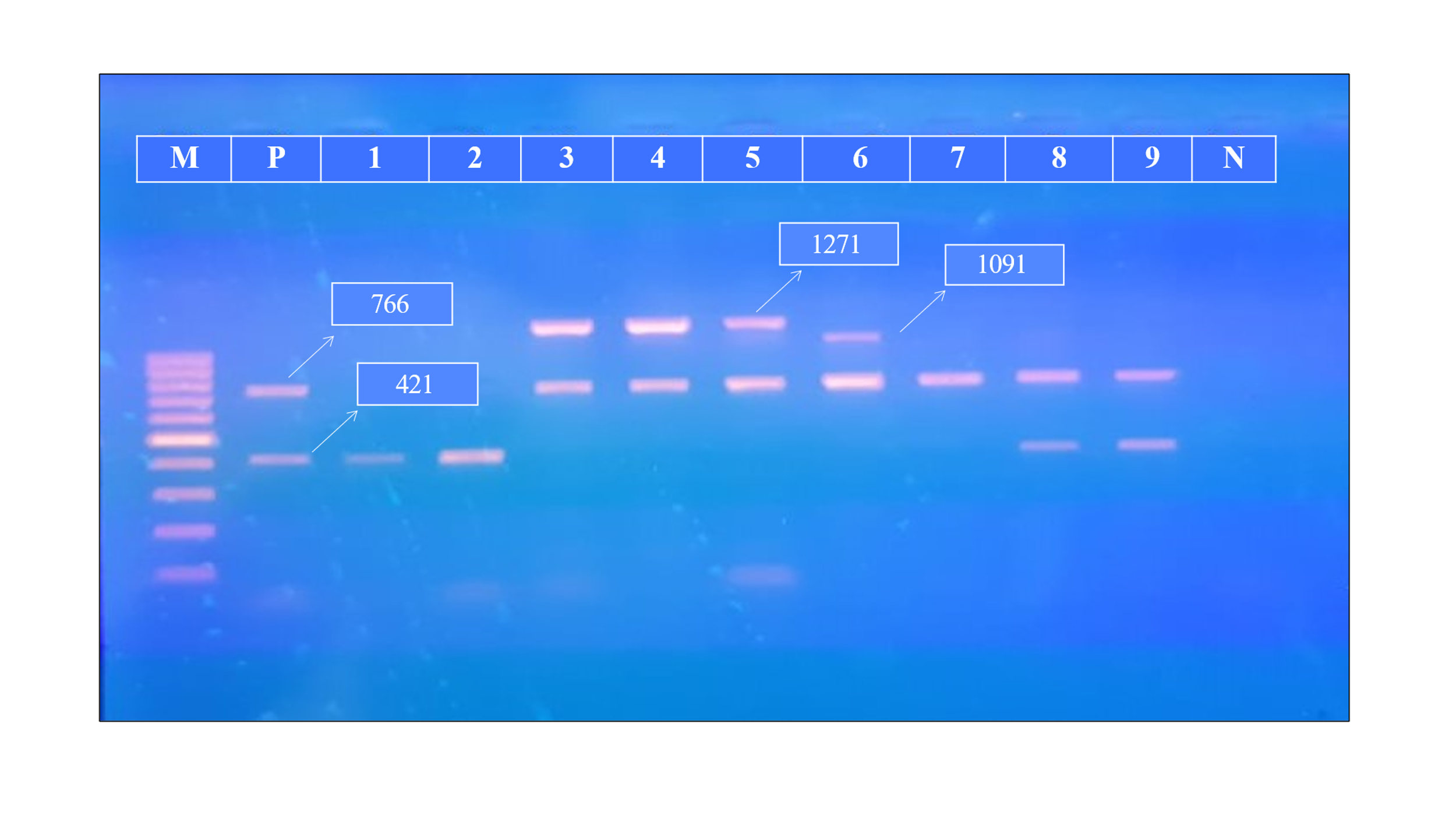


**Fig. S3.** Agarose gel of multiplex PCR products of *cytK* (421bp), *nhe* (766bp), *hbl* (1091bp) and *Ces* (1271bp)

M: molecular size marker (100bp DNA ladder), P*:* Positive control of *Bacillus cereus* (ATCC® 14579^TM^), Lines of 1, 2, 8, 9: represent positive isolates for *cytK* gene at 421bp, Lines of 3-9: represent positive isolates for *nhe* gene at 766bp, Line 6: represent positive isolate for *hbl* gene at 1091, Lines of 3-5: represent positive isolates for *Ces* gene at 1271bp, N: Negative control.
